# Bone mineral density and inflammatory bowel disease severity

**DOI:** 10.1590/1414-431X20176374

**Published:** 2017-10-19

**Authors:** C.A. Lima, A.C. Lyra, C.M.C. Mendes, M.B. Lopes, F.G. Coqueiro, R. Rocha, G.O. Santana

**Affiliations:** 1 Programa de Pós-Graduação em Medicina e Saúde, Universidade Federal da Bahia, Salvador, BA, Brasil; 2 Departamento de Gastroenterologia e Hepatologia, Universidade Federal da Bahia, Salvador, BA, Brasil; 3 Instituto de Ciências e Saúde, Universidade Federal da Bahia, Salvador, BA, Brasil; 4 Escola de Nutrição, Universidade Federal da Bahia, Salvador, BA, Brasil; 5 Departamento de Ciências da Saúde, Universidade do Estado da Bahia, Salvador, BA, Brasil

**Keywords:** Bone mineral density, Ulcerative colitis, Crohn’s disease, inflammatory bowel disease, Osteoporosis

## Abstract

Inflammatory bowel disease (IBD) is associated with low bone mineral density (BMD). In this study, the association between disease severity and BMD in patients with IBD was evaluated. Associations between BMD and the Montreal classification, disease activity and drug therapy were also tested. A cross-sectional prevalence study with a comparison group was conducted. One hundred and twenty-eight patients were evaluated: 68 patients with ulcerative colitis (UC), and 60 with Crohn's disease (CD). The control group consisted of 67 healthy subjects. All patients and controls had BMD measured and in IBD patients, current medications, hospitalization, and disease location, extent and phenotype, according to the Montreal classification, were recorded. Multiple correspondence analysis was applied to evaluate categorical variables. In the CD group, most patients were diagnosed between 17–40 years of age. Ileocolonic and non-stricturing non-penetrating disease were the most frequent disease location and behavior, respectively. In UC patients, extensive colitis was the most frequent disease location. UC and CD patients were more likely to have osteopenia than controls (OR=14.93/OR=24.38, respectively). In the CD group, male patients, perianal disease, penetrating behavior and age at diagnosis >40 years were associated with low BMD. Taking azathioprine and infliximab also seemed to be associated with osteopenia. In the UC group, we observed an association between low BMD and male patients, left colitis, corticosteroid use and hospitalization. Disease activity was not associated with osteopenia or osteoporosis in CD and UC patients. Disease severity seems to be associated with osteopenia in IBD patients.

## Introduction

Inflammatory bowel disease (IBD), including ulcerative colitis (UC) and Crohn's disease (CD), is associated with low bone mineral density (BMD). Prevalence of osteopenia and osteoporosis in IBD patients ranges from 22–77 and 17–41%, respectively, depending on the study population, study design and disease location (1).

Reduction in BMD is associated with increased risk of fractures. IBD patients can have up to 40% more fractures than the general population, which contributes to increased morbidity and reduced quality of life (2). Some studies have shown a higher BMD prevalence in CD patients than in those with UC (3), but this difference was not observed in other studies.

The exact bone loss mechanism is not well established. Initially, bone loss was attributed to the use of medications such as corticosteroids. However, studies in IBD patients without any treatment have shown low BMD in those patients, suggesting that the inflammatory process contributes to this mechanism (4). Several proinflammatory cytokines such as interleukin (IL)-1, tumor necrosis factor alpha (TNF-α), IL-6, IL-11, IL-15, and IL-17 are elevated in IBD and have been identified as stimulators of osteoclastogenesis (5).

Older age, smoking, physical inactivity and menopause are some known risk factors for osteoporosis in the general population and they may also be present in IBD patients. Other characteristics specific for IBD may increase the likelihood of osteoporosis and fractures like malnutrition, vitamin D deficiency, intestinal resection, and corticosteroids use (6).

Time from diagnosis and disease activity may be associated with lower BMD values (7). Some medications used to treat IBD seem to interfere with BMD. Use of corticosteroids is well established as a risk factor for osteoporosis, while azathioprine and anti-TNF therapy appear to contribute to increase bone mass (8,9).

The aim of this study was to evaluate association between disease severity and BMD in IBD patients. Another objective was to identify if there is an association between BMD and the Montreal classification, disease activity and drug therapy in these patients.

## Patients and Methods

A cross-sectional prevalence study with a comparison group was conducted with patients from two IBD treatment referral centers in Bahia, Brazil, both of which are located in the city of Salvador.

IBD patients, aged between 18 and 60 years, were included. Diagnosis of CD or UC was based on clinical, endoscopic, radiological and histological data.

Exclusion criteria were pregnancy, diseases that change bone metabolism (such as chronic renal failure, chronic obstructive pulmonary disease, thyroid disease, liver disease and systemic lupus erythematosus), cancer, diabetes mellitus, and women after menopause or use of estrogen therapy.

The control group was matched according to age and gender of UC and CD patients and comprised healthy volunteers recruited from the hospital staff, medical and nutrition students and patient’s relatives. The same exclusion criteria were used. None of them was taking medications known to affect bone turnover, and none had metabolic bone disease or had undergone intestinal resection.

Gender and age (years) from all participants were analyzed at time of inclusion. Time of IBD diagnosis, previous history of intestinal resection, current or last year of steroid use and cumulative dose, current medications, hospital admission in the previous year (due to disease activity), location, extent and phenotype of the disease, according to the Montreal classification (10) were also analyzed. Disease activity was assessed by the Harvey and Bradshow index (11) for CD and according to Lichtiger et al. (12) for UC.

Written informed consent was obtained from all patients and controls.

### Measurement of bone mineral density

Bone mineral density was measured by dual-energy X-ray absorptiometry (DEXA) using a Hologic QDR1000 densitometer (GE Medical Systems, USA). Measurement sites were femoral neck and lumbar spine. Results are reported as g/cm^2^ and presented either as Z-score or as a T-score.

According to the Guidelines of World Health Organization (WHO), T-score was used to determine low bone mineral density. Data were considered to be normal when they were within 1 standard deviation (SD), while osteopenia was defined as values from –1 SD to –2.5 SD and osteoporosis was defined as values equal to or below –2.5 SD compared to the normal population mean (WHO 1994) (13).

### Statistical analysis

Data were analyzed using Statistical Package R (version 3.1.1, 2010; Austria) (14). The population was characterized using descriptive statistics. Because the population of two reference centers was examined (rather than a random sample), statistical inference was not performed (15). For multivariate analysis, considering level of measurement of variables and multifactorial context, we chose the multiple correspondence analysis technique with eigenvalues decomposition Burt matrix adjusted for inertia and the standard coordinates multiplied by the masses for the evaluation of geometrical relationships in chi-square distances from the contingency of the variables studied in the multidimensional context (16) built from prior knowledge of literature and experience of the researcher, separately for ulcerative colitis and Crohn's disease considering its severity criteria.

### Ethical considerations

This study was approved by the Ethics Committee of the University Hospital Professor Edgard Santos, Salvador, Bahia, Brazil.

## Results

One hundred and twenty-eight patients were studied, 68 patients with UC (53.1%), and 60 with CD (46.9%). Age ranged from 19 to 56 years, mean 37.8±8.65 (SD) years. Most patients were female (56.3%). The control group consisted of 67 healthy subjects. No significant differences were found between patients and controls with respect to age and gender.

Most patients in the CD patients group (78.3%) were diagnosed between 17–40 years of age. Ileocolonic disease (L3 of the Montreal classification) was present in 32 patients (53.3%) and 29 patients (48.3%) had non-stricturing non-penetrating disease (B1 of the Montreal classification). Perianal disease was present in 20 patients (33.3%).

Most CD patients had not undergone surgery (64.2%). In those operated (19/60), partial colon resection was the most frequent surgery (63.2%), followed by small intestine and colonic resection (26.3%), and finally only small intestine resection (10.5%). Perianal surgery has not been evaluated.

Twenty-nine patients (44.6%) had extensive UC according to the Montreal classification. Only 2 patients had undergone surgery, 1 of whom underwent total proctocolectomy with ileal pouch. Baseline characteristics of patients and controls are reported in Table 1.

**Table 1.** Baseline characteristics of patients and controls.

	Crohn's disease (n=60)	Ulcerative colitis (n=68)	Controls (n=67)
Gender (female/male)	30/30 (50/50%)	42/26 (61.8/38.2%)	40/27 (59.7/40.3%)
Age (mean±SD)	37.4±8.3	38.2±9.0	36.3±8.67
Active disease	16 (26.7%)	2 (2.9%)	
Surgery	19 (31.7%)	2 (2.9%)	
Corticosteroid use	8 (13.3%)	12 (17.6%)	
Age at diagnosis			
<17 years	4 (6.7%)		
17–40 years	47 (78.3%)		
>40 years	9 (15%)		
Location			
L1 - ileal	8 (13.3%)		
L2 - colonic	19 (31.7%)		
L3 - ileocolonic	32 (53.3%)		
L4 - upper gastrointestinal	1 (1.7%)		
Behavior			
B1 - Non-stricturing, non-penetrating	29 (48.3%)		
B2 - Stricturing	13 (21.7%)		
B3 - Penetrating	18 (30%)		
p-perianal disease	20 (33.3%)		
Ulcerative proctitis		12 (17.6%)	
Left-sided ulcerative colitis		27 (39.7%)	
Extensive ulcerative colitis		29 (42.6%)	

The Montreal classification was used for evaluations.

Most of the CD patients were taking azathioprine (37, 61.7%), 11 patients were taking infliximab (18.3%), and only 8 patients received corticosteroid therapy in the past year (13.3%) with a mean cumulative dose of 1890±1017 mg, ranging from 330 to 3285 mg, and average duration of 4.5±3.2 months, ranging from 2 to 12 months. In UC patients, 88.2% were taking aminosalicylates, 8 patients were taking azathioprine (11.8%), and 12 (17.6%) used corticosteroid in the past year with a mean cumulative dose of 2072±1437 mg.

Most UC patients (97.1%) and CD patients (73.3%) were in clinical remission. Among the total sample, 28 UC (41.2%) and 32 CD patients (53.3%) had abnormal BMD, and osteoporosis was found in 2 UC patients (2.9%) and 7 CD patients (11.7%). In the control group, no one presented with osteoporosis and osteopenia was present in only 3 subjects (4.5%).

UC and CD patients were more likely to have osteopenia than controls (OR=14.93/OR=24.38, respectively).

**Figure 1.**
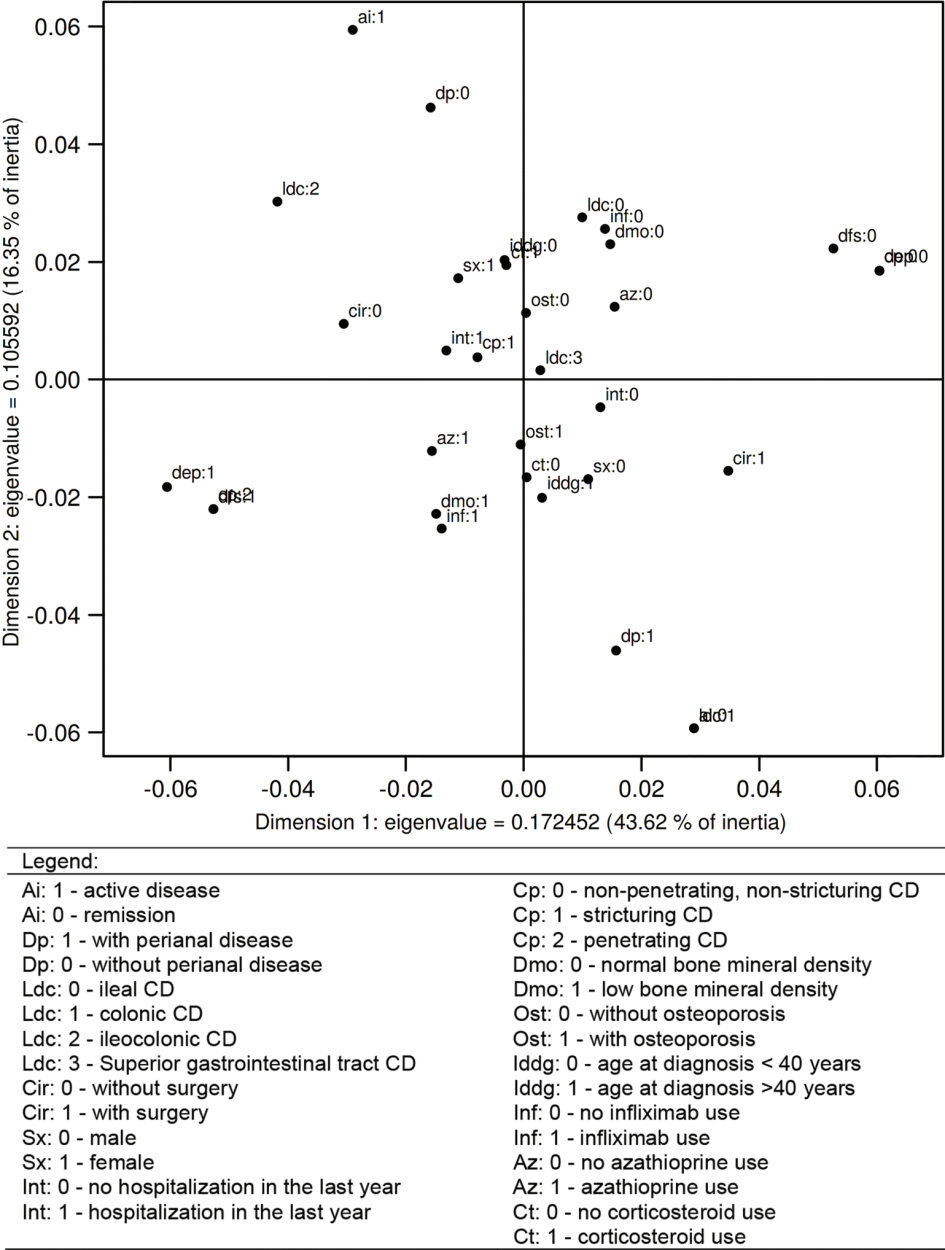
Multiple correspondence analyses for categorical variables in Crohn's disease (CD) patients.

According to multiple correspondence analysis, in CD group, male patients, perianal disease, penetrating behavior and age at diagnosis >40 years were associated with low BMD. Taking azathioprine and infliximab also seem to be associated with osteopenia. However, there was no association between BMD and hospitalization in the last year, nor surgery and disease activity (Figure 1).

**Figure 2.**
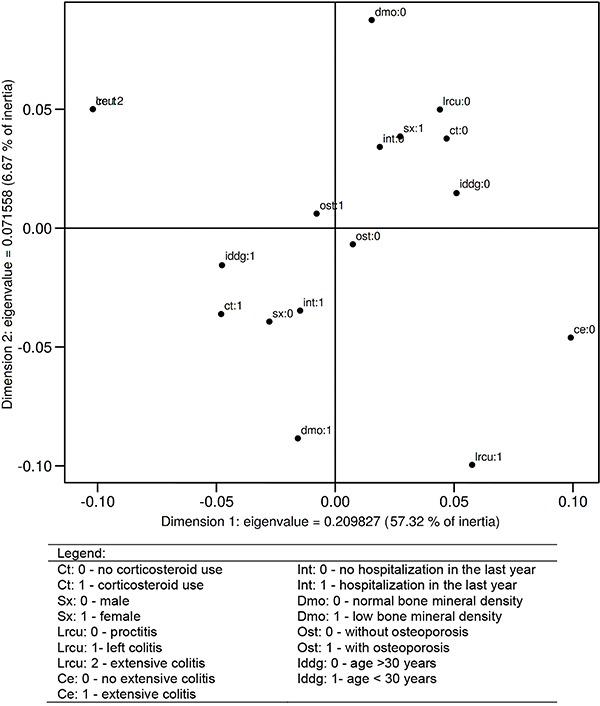
Multiple correspondence analyses for categorical variables in ulcerative colitis patients.

In the UC group, we observed an association between low BMD and male patients, left colitis, corticosteroid usage and hospitalization. Extensive colitis was not associated with osteopenia and osteoporosis (Figure 2).

## Discussion

IBD is associated with low BMD. In this study, a frequency of approximately 40% for osteopenia, and 7% for osteoporosis in IBD patients was found. Many deleterious factors are associated with bone loss in IBD patients. However, the exact mechanism associated with bone loss is not yet fully understood, as well as its associated risk factors. Studies of possible variables involved in this complication are crucial to prevent disabling diseases.

Similar frequencies of low BMD between UC and CD patients were observed in this study. However, Jahnsen et al. (3) showed that CD patients had lower BMD than UC patients and controls. Ezzat et al. (17) also demonstrated a higher frequency of low BMD in CD patients than UC patients. CD and vitamin D deficiency were predictors of osteopenia and osteoporosis in their study. But, in these 2 studies, CD patients with lower BMI had used higher doses of corticosteroids than UC patients, which may have affected the results. Other authors also did not demonstrate this difference. Bjarnason et al. (18) studied 44 CD patients and 35 UC patients. Disease type, location and severity of IBD were not related to BMD reduction. Sakellariou et al. (4) also found no difference between IBD type and BMD in steroid-naive young male patients.

There is still no definition whether gender affects BMD in IBD patients. Ardizzone et al. (19) studied 91 IBD patients (51 with CD and 40 with UC). BMD of spine and femur, T-score and z-score were significantly lower in male than in female UC patients, but in CD patients no difference was observed. Andreassen et al. (20) have shown that female gender was a predictor of low BMD in CD patients, as well as age and weight. Our results were similar to the Italian study (19), which observed low BMD in male UC patients. In our study, male CD patients were also associated with low BMD. Szathmári et al. (21) studied 45 men with IBD and reported that 23 patients had low levels of dehydroepiandrosterone sulphate (DHEAS) and a correlation between DHEAS levels and the BMD in lumbar spine and femoral neck. One possible reason for this finding is that IBD male patients have lower DHEAS levels, which may have contributed to bone loss.

There is a lack of consistent definition of aggressive and disabling UC. Some authors have defined aggressive UC as those patients who need immunosuppressive therapy or surgery. Few factors have been related with an aggressive course of UC like age at diagnosis, corticosteroid use at diagnosis, extensive disease and history of hospitalization. A retrospective case-control study with 246 UC patients found that requiring medical hospitalization for management of UC (odds ratio, OR=5.37, 95% confidence interval (CI)=2.00–14.46) and requiring infliximab therapy (OR=3.12, 95%CI=1.21–8.07) were independent predictors of colectomy (22). However, Leijonmarck et al. (23) studied factors affecting colectomy rate in a retrospective population-based series of 1586 patients with ulcerative colitis. They reported that the main factor affecting colectomy rate was disease extension. In a multicenter analysis of 262 UC patients, Stallmach et al. (24) identified 6 independent clinical parameters (age at diagnosis, gender, necessity of steroids or hospitalization at diagnosis, extent of the disease and result of an initial steroid therapy) that can be used to establish a prognostic model to predict the individual risk of requiring an immunosuppressive therapy. Extensive colitis and/or hospitalization during the last year were criteria applied to identify severe UC patients in our study.

There is also no definition of disabling CD. Stricturing or penetrating disease behavior is considered a complicated disease. Beaugerie et al. (25) arbitrarily defined CD disabling course when presenting with at least one of the following criteria: having received more than 2 courses of corticosteroids and/or corticosteroid dependence, hospitalization because of disease relapse, development of CD complications, presence of disabling symptoms for more than 12 months during a 5-year follow-up period, requirement of therapy with immunosuppressive agents, intestinal resection, or operation because of perianal disease. In that study, they reported that age below 40 years, presence of perianal disease, and initial requirement for steroids were predictors of disabling CD. In an American population-based cohort, Thia et al. (26) evaluated evolution of CD behavior applying the Montreal classification, and determined predictors of fistulizing or stricturing complications. In that study, patients with ileal disease had a 9-fold increased risk (HR=9.25; 95%CI=4.10–20.87, P<0.001) to develop an intestinal complication relative to those with colonic disease, while ileocolonic CD patients had a 6-fold increased risk (HR=5.74; 95%CI=2.33–14.13, P<0.001) to develop a complication. In our study, we considered as severe CD patients those with age at diagnosis below 40 years, presence of penetrating or stricturing behavior, perianal disease, ileal involvement and hospitalization during the last year.

Among UC patients, a higher frequency of extensive colitis and left colitis was found, while in CD patients, most (51.7%) had more severe disease phenotype (stricturing and penetrating CD). These findings may have occurred since the survey was conducted in 2 centers of excellence in IBD. In the CD patients group, no association between low BMD and disease location was observed. However, penetrating disease and age at diagnosis >40 years were associated with osteopenia. Vázquez et al. (27) conducted a prospective case-control study of 107 IBD patients and showed neither the extent nor the location of the disease had interfered significantly with BMD and presence of fractures, both in UC and CD patients. Cravo et al. (8) observed that stricturing and penetrating phenotype, small bowel disease, older age, history of surgery, and duration of illness over 15 years were associated with osteoporosis in CD patients. In the UC patients group, we found an association between left colitis and low BMD, but no association was evidenced between extensive colitis and osteopenia/osteoporosis. A study by Jahnsen et al. (3) with 60 UC patients, did not demonstrate interference of disease extension in BMD. Thirty-five UC patients were studied by Bjarnason et al. (18) and no difference in T-score was observed between proctosigmoiditis and extensive colitis.

In our study, we did not find association between BMD and hospitalization in the previous year in CD patients. However, hospitalization (due to managing of disease activity) was associated with low BMD in UC patients. Azathioprine and infliximab (IFX) use was associated with low BMD in CD patients. A Canadian study published by Targownik et al. (28) also found no association between history of hospitalization, duration of illness and use of medications with low T-score or risk of osteoporosis in IBD patients compared with controls. Florén et al. (29) analyzed the effect of azathioprine on bone density in a retrospective study with 59 CD patients. They found that azathioprine does not seem to affect bone mineral density by itself, but it seems to conserve bone mineral mass in CD patients by reducing corticosteroid use. Cravo et al. (8) studied mild to moderate CD patients (n=99) and found azathioprine to be a protective factor. In relation to IFX use, no association was observed in this study, although the number of patients using this medication was small. A retrospective study by Mauro et al. (30) showed a significant increase in BMD at the lumbar spine in CD patients treated with IFX when compared with control group (P<0.01). Pazianas et al. (31) had also shown benefit of using IFX in BMD in a retrospective cohort. They studied 61 CD patients and those who used bisphosphonates plus IFX experienced a greater increase in BMD than those using only bisphosphonate (+ 6.7%/year × 4.46%/year, P<0.050). In our study, few patients were on IFX therapy (n=11), which may have interfered in our results. Furthermore, we did not evaluate for how long patients were taking azathioprine and if their disease was really in remission, because the criteria used were those of Harvey and Bradshaw (11) for CD and Lichtiger et al. (12) for UC, that assess clinical remission but not endoscopic remission.

Many studies have identified association between use of systemic corticosteroids (CS) with osteoporosis and bone loss in IBD patients. Use of CS alters the balance between osteoblasts and osteoclasts. CS stimulate osteoclast differentiation and activation, and induce osteoblasts apoptosis, reducing bone formation (32,33). Abraham et al. (34) studied 166 IBD patients and showed that CS use more than doubled the risk of low BMD (OR=2.4 (1.5-3.6), P=0.001). Ezzat et al. (17) also observed a negative correlation between low BMD and cumulative CS dose and duration of its use. However, other authors did not find a clear association of low BMD with CS use. One hundred and thirteen CD patients were studied by Andreassen et al. (20) and no correlation between BMD and cumulative dose of CS was found. Miznerova et al. (35) studied 76 IBD patients and also no negative relationship between the corticosteroids use and BMD was found. Indeed, a positive correlation between the mean prednisone dose and BMD of the femur was observed, which may be due to the improvement of inflammation. In our study, we demonstrated an association between corticosteroids use and low BMD in UC patients, but not in CD patients. However, only 8 CD patients received corticosteroids in the previous year and had low cumulative dose (average of less than 2 g of prednisone).

Inflammation *per se* contributes to reduction of BMD. A number of inflammatory diseases have been associated with osteoporosis. A cross-sectional study by Bjarnason et al. (18) evaluated 79 IBD patients and observed that patients with newly diagnosed UC or CD and who had never used corticosteroids also had low BMD. The main system involved in bone loss in IBD is probably RANK/RANKL/osteoprotegerin, which promotes osteoclastogenesis (5,6). Several proinflammatory cytokines such as IL-1, IL-6, TNF-α, IL-11, IL-15 and IL-17 are associated with activation of osteoclasts and are also high in IBD (6). In our study, no association was found between disease activity and reduction in BMD. However, only 2 UC patients and 16 CD patients presented active disease at the time DEXA was performed, which may have affected the results as remission disease can be associated with increase in BMD. Reffitt et al. (36) conducted a study of 137 IBD patients and demonstrated a correlation between z-score increase and time of disease in remission in both UC and CD patients. Although it was a cross-sectional study and results were limited, this study provides evidence of an association between disease remission and greater bone density in both the lumbar spine and femoral neck.

Some studies have shown that surgery may be related to greater bone loss in IBD patients. Gupta et al. (37) studied a total of 126 IBD patients with ostomy, and most had CD and ileostomy. A prevalence of 29% of low BMD was found and presence of fractures was 5 times more common in these patients. Shen et al. (38) have conducted a case-control study of 327 UC patients who had undergone total proctocolectomy with ileal pouch. They found a prevalence of 32% of low BMD in these patients. In our study, no association was found between history of surgery and change in BMD. However, this result may be due to the small number of patients submitted to surgery and the type of surgery. Only one UC patient had undergone complete colectomy with ileal pouch and no CD patient had ostomy when DEXA was performed.

A cross-sectional study by Lora et al. (39), with a total of 76 IBD patients and 40 controls from southern Brazil that was similar to ours, found low BMD in IBD patients, especially in CD patients, but no association was identified between BMD and corticosteroids use, surgery and disease activity. Although done in a different location than our study and with fewer patients, similar results were found, as we also did not find association between some of these clinical criteria and low BMD.

In our study, we found lower a BMD in IBD patients compared with controls. Low BMD was associated with penetrating and perianal disease, age at diagnosis >40 years and male patients in CD group. In the UC group, male patients, left colitis, corticosteroid use and hospitalization were associated with osteopenia. Therefore, disease severity seems to be associated with osteopenia in IBD patients. Disease activity was not associated with low BMD, while azathioprine and infliximab use were associated with osteopenia. Prospective studies are needed to better define the relationship between these drugs and BMD.
